# Quantum advantage for probabilistic one-time programs

**DOI:** 10.1038/s41467-018-07591-2

**Published:** 2018-12-06

**Authors:** Marie-Christine Roehsner, Joshua A. Kettlewell, Tiago B. Batalhão, Joseph F. Fitzsimons, Philip Walther

**Affiliations:** 1 Vienna Center for Quantum Science and Technology, Faculty of Physics, University of Vienna, Boltzmanngasse 5, 1090 Vienna, Austria; 2 Singapore University of Technology and Design, 8 Somapah Road, Singapore, 487372 Singapore; 3 Centre for Quantum Technologies, National University of Singapore, 3 Science Drive 2, Singapore, 117543 Singapore; 4 Centro de Ciências Naturais e Humanas, Universidade Federal do ABC, Avenida dos Estados 5001, 09210-580 Santo André, São Paulo Brazil; 5 Erwin Schrödinger International Institute for Mathematics and Physics, University of Vienna, 1090 Vienna, Austria

## Abstract

One-time programs, computer programs which self-destruct after being run only once, are a powerful building block in cryptography and would allow for new forms of secure software distribution. However, ideal one-time programs have been proved to be unachievable using either classical or quantum resources. Here we relax the definition of one-time programs to allow some probability of error in the output and show that quantum mechanics offers security advantages over purely classical resources. We introduce a scheme for encoding probabilistic one-time programs as quantum states with prescribed measurement settings, explore their security, and experimentally demonstrate various one-time programs using measurements on single-photon states. These include classical logic gates, a program to solve Yao’s millionaires problem, and a one-time delegation of a digital signature. By combining quantum and classical technology, we demonstrate that quantum techniques can enhance computing capabilities even before full-scale quantum computers are available.

## Abstract (web summary)

Information Theoretically-secure deterministic programs that self-destruct after a single use are known to be impossible to implement. Here, the authors use quantum states to implement a probabilistic version of this fundamental cryptographic primitive, and provide a proof-of-principle implementation with single photons.

## Introduction

With the continuous march of technological advancement, computer processors have become ubiquitous, impacting almost every aspect of our daily lives. Whether being used to compose email or acting as control systems for industrial applications, these devices rely on specially written software to ensure their correct operation. In many cases it would be desirable to prevent a program from being duplicated or to control the number of times a program could be executed, for example, to prevent reverse-engineering or to ensure compliance with licensing restrictions. Unfortunately, the very nature of classical information ensures that software can in principle always be copied and rerun, enabling various misuses.

As a solution to this and other problems, the concept of one-time programs was introduced^1^. One-time programs are a computational paradigm that allows for functions that can be executed one time and one time only. Thus, if a software vendor encodes a function *f* as a one-time program, a user having only one copy of that program can obtain only one input–output pair (*x*, *f*(*x*)) before the program becomes inoperable. In the classical world, this is only possible through the use of one-time hardware or one-time memories^1^, special-purpose hardware that gets physically destroyed after being used once. However, it is unclear whether such hardware can be realised in an absolutely secure way. An adversary may attack the specific implementation, seeking to circumvent or reverse whatever physical process is used to disable the device after a single use.

Certain features of quantum mechanics, such as the no-cloning theorem^2,3^ and the irreversibility of measurements^4^, suggest that it may enable a solution to this problem. It was recently shown, however, that deterministic one-time programs are impossible even in the quantum regime^5^. As a result, it is believed that neither classical nor quantum information theoretically secure one-time programs are possible^1,5–9^ without further assumptions^10–14^.

Here, we demonstrate theoretically and experimentally that quantum mechanics does enable a form of probabilistic one-time program which shows an advantage over any possible classical counterpart. These rely on quantum information processing to execute, but encode entirely classical computation. Such probabilistic one-time programs circumvent existing no-go results by allowing a (bounded) probability of error in the output of the computation. We show that these quantum one-time programs offer a trade-off between accuracy and number of lines of the truth table read, which is not possible in the classical case. Remarkably, the experimental requirements to encode the probabilistic one-time programs we introduce are comparable to those of many quantum key distribution implementations, allowing for technological advances in that field to be harnessed for a new application.

## Results

### Construction of the one-time programs

**Fig. 1:**
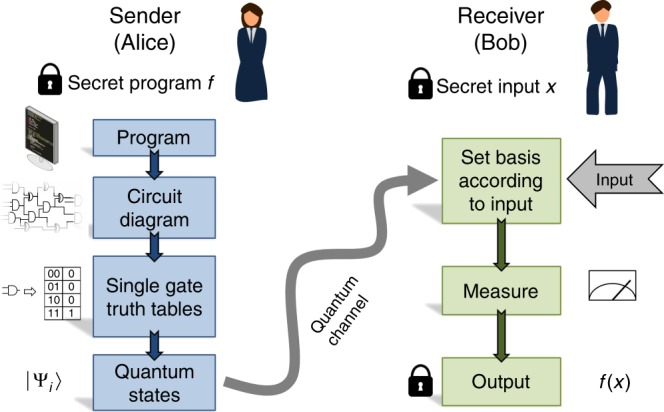
Overview of a probabilistic one-time program scheme. Alice possesses a secret program, *f*, and Bob a secret input, *x*. Alice converts *f* into a logic circuit. Next, Alice encodes the logic gates comprising the circuit as non-orthogonal quantum states. For the particular encoding scheme we realise experimentally, these are always separable states. These states are sent to Bob via a quantum channel. Bob executes the program by sequentially measuring the quantum states corresponding to individual logic gates. The basis for each measurement is determined by Bob’s input to that gate and the measurement result represents the output of the gate, up to some bounded probability of error. The encoding can be chosen such that it suffices for Bob to make only single-qubit measurements. Intuitively, the security of the scheme stems from the fact that the measurements corresponding to different inputs for a given gate do not commute, which prevents Bob from evaluating more than one input

We consider one-time programs (OTPs) in the context of a two-party setting, where Alice is the software provider and Bob is the user. Alice’s program is represented by a secret function *f*, which she encodes as a separable state of some number of qubits, which scales linearly in the number of elementary logic gates required to implement *f*, and provides these to Bob. Bob can then evaluate *f* on some input of his choice *x* by sequentially measuring each qubit received from Alice. These measurements are a fundamentally irreversible process, which is necessary for Bob to evaluate *f*(*x*) while at the same time preventing him from learning *f*(*x*′) for some input *x*′ ≠ *x*. An outline of our approach is presented in Fig. 1.

**Fig. 2:**
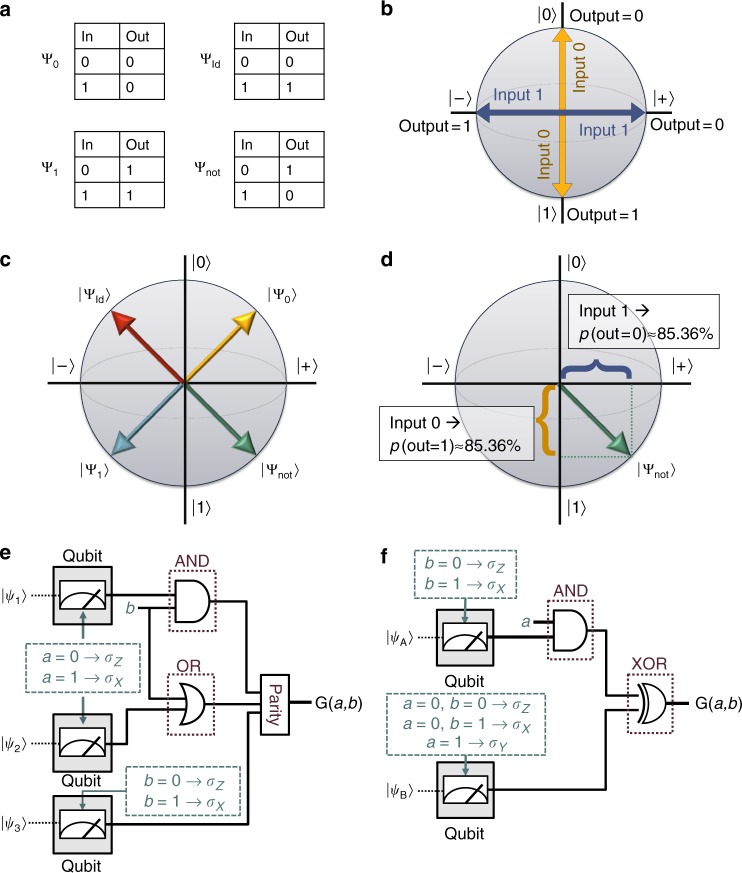
Method for constructing probabilistic one-time programs for gates with one and two bits of input. All possible truth tables for gates in ${\cal G}_1$ are shown in (**a**) while (**b**) shows the encoding of Bob’s inputs: a measurement in *σ*_*Z*_ corresponds to an input of 0 to the gate while a measurement in *σ*_*X*_ corresponds to an input of 1. The output of the gate is given by the measurement outcome. **c** An overview of the four ${\cal G}_1$ states in a Bloch-sphere representation. A detailed example is shown in (**d**): when measured in *σ*_*Z*_ (i.e., input 0) the state corresponding to Ψ_not_ will be found in the state |1〉, corresponding to an output of 1, with a probability of about 85.36%. When measured in *σ*_*X*_ (i.e., input 1) the measurement will find |+〉, so an outcome of 0, with the same probability. **e**, **f** Two equivalent circuits to build an arbitrary ${\cal G}_2$ gate. The (**e**) is based on three ${\cal G}_1$ gates while the circuit shown in (**f**) only requires two quantum states per gate, some of which however need to be outside the *X*–*Z* plane

In analogy to the compiling of standard classical programs, the logic of *f* is mapped onto a logic circuit using basic logic synthesis^15^. It is necessary that the circuits have a certain standard form, such that the information to be hidden is encoded in the precise choice of logic gates and not on the connections between gates. This is because our approach is to encode the truth table for individual gates as a one-time program in its own right, which we will call gate one-time programs (gate-OTPs). The interconnection of gates is left public, allowing Bob to propagate information from one gate to the next. Each logic gate is a Boolean function, taking *k* input bits and returning a single output bit. We will denote the set of *k*-input gates as ${\cal G}_k$. For *k* ≥ 2, it is possible to implement an arbitrary Boolean function on *n* input bits with gates chosen only from ${\cal G}_k$ together with the fan-out operation^16^ that defines the number of output bits. It is however possible to build up arbitrary ${\cal G}_k$ gates from a fixed configuration, with some choice of gates from ${\cal G}_1$. Such a construction of an arbitrary ${\cal G}_2$ gate is shown in Fig. 2e.

Probabilistic versions of the four gates comprising ${\cal G}_1$ can be encoded using a single qubit, as shown in Fig. 2a–d, such that the measurement operators corresponding to different inputs anti-commute. This is achieved by first fixing the measurement bases corresponding to inputs of 0 and 1 respectively (Fig. 2b), and then finding the states to encode each gate such that it maximises the average probability of obtaining the correct outcome across both possible inputs (Fig. 2c). The measurement bases are chosen to be unbiased and correspond to anti-commuting observables, *σ*_*Z*_ for input 0 and *σ*_*X*_ for input 1, to ensure that in learning about the value of one observable Bob must forego information on the other. Once the measurement bases are fixed, the states can be found which yield the correct output with a maximal probability of $\frac{1}{2} + \frac{1}{{2\sqrt 2 }}$ or approximately 85.36%. This encoding relates to conjugate encoding introduced by Wiesner^17^ and is equivalent to the quantum random access codes considered in ref. ^18^, which were motivated by ideas of compression rather than security. However, the concepts of one-time programs and random access codes diverge when we consider hiding gates from ${\cal G}_k$ for *k* > 1 later on.

With a method for implementing ${\cal G}_1$ now in place, we can proceed to construct a universal set of gates, for example ${\cal G}_2$, while preventing Bob from learning the full truth table. As alluded to previously, one way to achieve this is to insert hidden ${\cal G}_1$ gates into a fixed circuit, as shown in Fig. 2. The exact choices required for each of the hidden gates to achieve a specific ${\cal G}_2$ gate is described in the Supplementary Tables 1 and 2. The overall success probability for gates constructed in this way is 75%. However, such an approach yields a rather complicated construction for gates in ${\cal G}_k$ for *k* > 2 and introduces complications in the security analysis. A more appealing approach is to directly implement probabilistic one-time programs for gates in ${\cal G}_k$. This can be done by generalising the construction used in the *k* = 1 case. Specifically, each possible input is assigned a unique observable from a set of anti-commuting multi-qubit Pauli operators {*σ*_*i*_}, where a + 1 measurement outcome is taken to correspond to a gate output of 0 and a − 1 outcome is taken to correspond to an output of 1. As before, the states encoding each gate *G* are chosen to maximise the average probability that the outcome of measuring the observable corresponding to input *x* results in output *G*(*x*). Unlike the case for ${\cal G}_1$, there is an entire subspace of states satisfying this constraint for a given *G*. Our approach is to encode *G* as the maximum entropy state maximising success probability,

1 $$\rho _G = \frac{1}{{{\mathrm{tr}}({\Bbb I})}}\left( {{\Bbb I} + \frac{1}{{\sqrt {2^k} }}\mathop {\sum}\limits_{i = 0}^{2^k - 1} \left( { - 1} \right)^{G(i)}\sigma _i} \right).$$

This coincides with the definition of a particular type of random access code, known as a parity oblivious random access code, explored in ref. ^19^ for other purposes. The success probability for any input *i* is then given by $\frac{1}{2}\left( {1 + ( - 1)^{G(i)}{\mathrm{tr}}(\rho _G\sigma _i)} \right)$ which simplifies to $\frac{1}{2}\left( {1 + 2^{ - \frac{k}{2}}} \right)$. Remarkably, this results in each *ρ*_*G*_ being the maximally mixed state of a 2^*k*−1^-dimensional subspace, so that the von Neumann entropy is *k* − 1 bits.

The implementation of ${\cal G}_k$ gates requires 2^*k*^ anti-commuting operators and 2^*k*−1^ qubits. However, there is an alternative implementation that uses 2^*k*^ − 1 qubits whose Pauli operators are restricted to being tensor products of the identity, *σ*_*X*_ and *σ*_*Z*_. While there is no fundamental reason to require such a restriction, it can reduce the hardware requirements necessary to implement the scheme, as seen in the experimental section. An explicit construction in terms of separable states for the case where *k* = 2 is given in the the Methods section and Supplementary Tables 1 and 2, both with and without this restriction. These encodings form the basis for the experimental implementations with linearly and elliptically polarised photons respectively.

### Experimental implementation

**Fig. 3:**
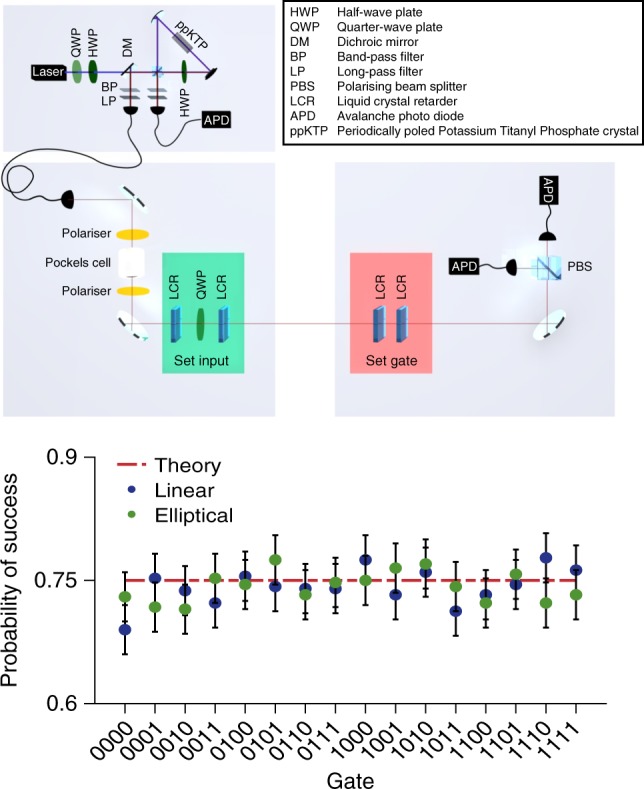
Experimental one-time program implementation. **a** Our setup: marked in green are the liquid crystal retarders (LCRs) and quarter-wave plate (QWP) used to manipulate the polarisation state of single photons which corresponds to setting the individual gates of the encoded program. The LCRs marked in red are used by Bob to set the measurement basis according to the gate inputs. On Alice’s side an active switch is implemented based on a Pockels cell placed between two crossed polarisers acting as a fast switching half-wave plate. Alice produces single photons with a source in a Sagnac configuration^28–30^. In (**b**) we show the average success probability per gate for all ${\cal G}_2$ gates. Blue dots represent the results for the linear scheme, green dots the results for the elliptical scheme and the theoretically predicted value is shown by the red line. The gates are labelled by the last column of their truth table, e.g. (00 → 0, 01 → 1, 10 → 0, 11 → 0) corresponds to 0100. Error bars show an interval of ±1 standard deviation derived from binomial statistics and are therefore a lower bound

To demonstrate the viability of the presented scheme, we show a proof-of-principle implementation based on polarisation encoded photonic qubits (Fig. 3a; see Methods for details).

We realised two equivalent schemes: we refer to the first one as the linear scheme because it can be implemented using only linearly polarised photons. This version requires fewer technological resources: Alice and Bob each need just one liquid crystal retarder (LCR). These LCRs rotate the polarisation of each photon by an angle depending on the applied voltage and are therefore used to actively switch from one polarisation setting (corresponding to a gate or a measurement basis) to the next. However, in this encoding three photons per ${\cal G}_2$ gate are required. Our elliptical scheme uses elliptically polarised states and requires two LCRs per party. The advantage of this scheme is that it only requires two photons per ${\cal G}_2$ gate, reducing the length of the program by a third.

For both versions we tested all 16 gates comprising ${\cal G}_2$ for all four possible inputs (00, 01, 10, 11). The average success probability of each gate is shown in Fig. 3b, and the results are in good agreement with the expected value of 0.75. We characterised all single-photon states using quantum state tomography^20^ where a fidelity, *F* ≥ 0.991 ± 0.008 could be achieved for all states (see Supplementary Table 3 for details).

### Demonstrated programs

To demonstrate the applicability of our scheme we have experimentally implemented two different classes of one-time programs.

**Fig. 4:**
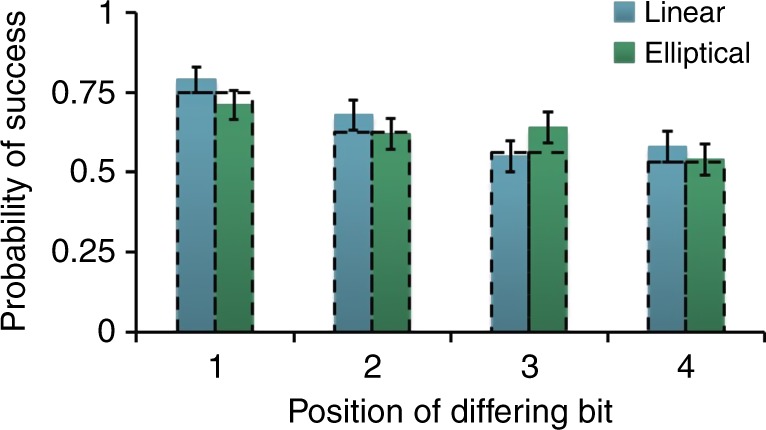
Success probability for the millionaires problem using the linear (blue or first bar) and elliptical (green or second bar) scheme. We compare different four-bit numbers in binary representation to the number 0101, where each compared number deviates from 0101 in exactly one bit. The expected probability of success (shown in black dotted lines) depends on the position of the differing bit (1 corresponds to the most significant bit, 4 to the least significant bit). Error bars show an interval of ±1 standard deviation derived from binomial statistics and are therefore a lower bound

The first class we consider is a program built from a combination of ${\cal G}_2$ gates which are universal for classical computation. We use it to solve Yao’s millionaires problem^14^, in which two people wish to compare their wealth without disclosing this value to the other party. To accomplish this goal, Alice encodes her wealth into the program. Bob’s wealth will be his input (see Supplementary Figure 1). The program returns a single bit, indicating which number is larger. We ran the millionaires problem using both the linear and the elliptical schemes on several inputs. Alice encoded a four-bit number and Bob compared it to numbers that each differed in one bit from Alice’s input. The detailed results are shown in Fig. 4. In good agreement with our theoretical expectations, it can be seen that the probability of success rises with the significance of the bit in which the two numbers differ (i.e., it is easier to discriminate two numbers that differ in the most significant bit than two that differ in the least significant bit).

The second kind of program we consider concerns the delegation of digital signatures or one-time power of attorney. Here Alice can enable Bob to sign one, and only one, message of his choice with a signature derived from her private key. However, due to the probabilistic nature of the described OTPs, there is a non-negligible probability that OTPs will not output the correct signature. To compensate for this we may repeat the procedure and define some threshold number of signatures which is announced publicly to be an acceptable number required to verify a given message has been signed. Alice produces many distinct OTPs each using a different private key such that Bob has a high probability of forming the required number of signatures for a single message.

**Fig. 5:**
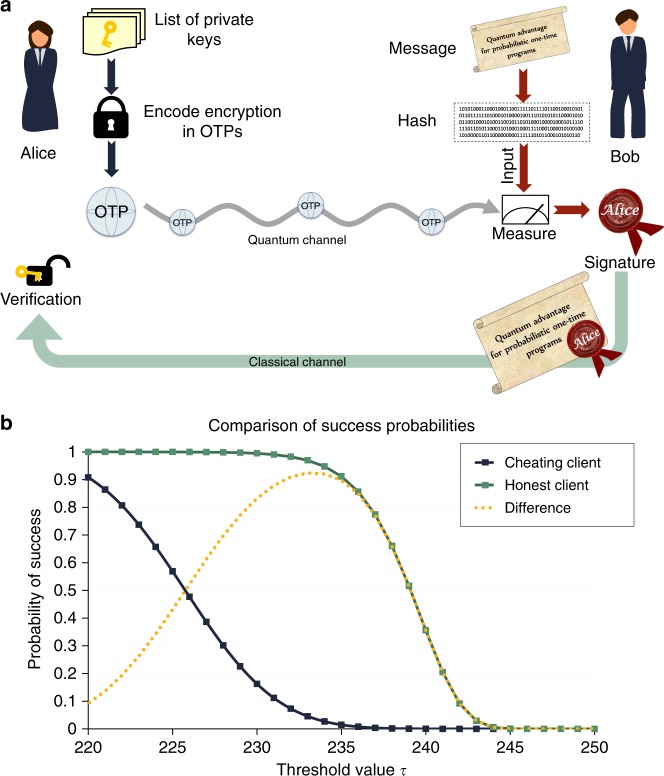
Private key one-time signature scheme. **a** Overview of the private key one-time signature scheme. Alice encodes her signature program as noisy quantum OTPs, and sends them to Bob via a quantum channel. Bob measures the states according to the hash of a message he wishes to sign, with outputs corresponding to a signature. This message and signature pair may be verified by Alice at a later stage. **b** Bob’s success probabilities of signing a message or messages, in both the honest (one message) and the dishonest (two messages) cases. The probability of the client successfully generating a valid signature is plotted against the threshold value for number of correct bits required to sign a message, given that the client is provided with 300 OTPs for each row of the table. The green line shows the probability of an honest client generating a single signature (≥*τ* correct outputs for each row of the signature), as a function of the threshold number of correct bits required in each row. The blue line shows probability of a dishonest client generating two signatures, which differ only by one bit in the hash (the probability of a client following an honest strategy in all but one row). The difference between the probabilities of the prior two lines is indicated by the yellow line. Details are given in the Supplementary Note 4—Description of the Private Key Signature scheme

Standard signature schemes use a public key for verification and are widely used within cryptographic protocols to guarantee authentication, non-repudiation and integrity^21–24^. However, due to technical reasons limiting our gate rate in experiment, we restrict our demonstration to a symmetric digital signature scheme, wherein Alice’s private key is used to verify a signature. Such a program may be utilised for a third party to spend an amount of money on someone else’s behalf, so that they should pay anyone with a signed receipt. An overview of this scheme is shown in Fig. 5a. Bob computes a hash of the message he wishes to sign and uses this as the input to the OTPs (using a hash ensures that the input length does not depend on the length of the actual message signed). The output of the OTPs will then be the digital signature which Alice may verify. For each bit of this hash Alice provides e.g. 300 ${\cal G}_1$ gates, from which Bob produces a bit string dependent on the result of measuring according to that bit. Such a bit string may be compared by Alice to the ideal case where all gates have been implemented on the corresponding hash bit. We require that each bit string matches such an ideal string in at least *τ* positions to produce a valid signature. The threshold *τ* is chosen as a function of the bit string length *T* to maximise the difference between the probabilities of success of the honest and dishonest strategies, where in a dishonest strategy Bob would attempt to sign two hashes differing only by a single bit. This is illustrated in Fig. 5b and in Supplementary Figure 2. As *T* is increased, the probability of an honest Bob forming a sufficient fraction of correct bits (≥*τ*/*T*) in each bit string approaches 1, while that of a dishonest user who would try to form multiple signatures approaches 0. This demonstrates a clear example of a case where even probabilistic one-time programs enable new functionality that is inexecutable using classical technology.

### Security analysis

We will now discuss the security of our protocol and show a strict advantage over any possible classical strategy. We note that the security relies on several measures affecting different steps of our protocol. Starting with the logical synthesis we see that when gate-OTPs are combined into circuits, there is some freedom over how the gates are chosen. In our proof-of-principle demonstration of Yao’s millionaires problem we limit the information accessible to Bob by randomly inserting pairs of NOT gates into the circuit immediately after each gate-OTP with probability one-half. The first NOT gate is absorbed backwards into the gate-OTP, altering the encoded gate. The second NOT of the pair is propagated forward, through any present fan-out and XOR gates, and absorbed into the next layer of gate-OTPs, altering the function they encode. Such a procedure can always be applied to any circuit composed of gate-OTPs along with XOR, NOT and fan-out operations.

To analyse the effect of this randomisation procedure, we will assume it is applied after every gate-OTP. In such a case, the joint state of the quantum systems used to encode the gate-OTPs is maximally mixed, and hence independent of the encoded function. For those gate-OTPs which produce the output of the program the second NOT gate cannot be absorbed into a subsequent gate-OTP. We will simply eliminate this second NOT gate, effectively applying a one-time pad to the program’s output and creating the maximally mixed state from the perspective of the receiver. Such a scheme thus negates all losses in the system as the maximally mixed state does not allow a dishonest user to extract any information regarding the intended gate-OTP. Since the output of the program can be revealed by decoding the one-time pad, the accessible information for the entire system can be no greater than the size of this encryption key, and hence can be no greater than the number of output bits for the program. This is in line with the requirement that a one-time program should reveal no more information than can be obtained from a single run of the program.

**Fig. 6:**
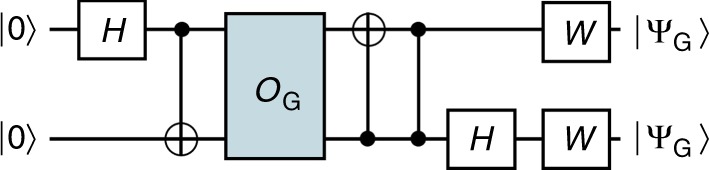
Security of probabilistic one-time programs. We demonstrate that the ability to make a single query to the (ideal) classical gate allows us to have 2 copies of state |*ψ*_*G*_〉. The quantum circuit shown in the figure produces two copies of the state |*ψ*_*G*_〉 for any $G \in {\cal G}_1$ using only a single query of *O*_*G*_ which is an implementation of a reversible version of the encoded gate *G* mapping |*x*, *y*〉 to |*x*, *y* ⊕ *G*(*x*)〉 and *W* = [*c*, *s*; *s*,−*c*], where *c* = cos(*π*/8) and *s* = sin(*π*/8). As it is not possible to produce two copies of |*ψ*_*G*_〉 from a single copy, this demonstrates strictly less can be learned from a single copy of output state ${\cal G}_k$, than from a single (coherent) query to the encoded function

We now consider the security of the individual gate-OTPs corresponding to gates in ${\cal G}_1$. We show that strictly less can be learned from a single copy of them than from a single query to the encoded function (i.e., an ideal one-time implementation of that function). For all gates $G \in {\cal G}_1$, the corresponding state *ρ*_*G*_ is pure, and so we will denote the state vector as |*ψ*_*G*_〉. Figure 6 shows how a single query of the encoded function can be used to produce two copies of this state. The fact that states encoding different programs are non-orthogonal, coupled with the no-cloning theorem^2,3^, implies it is not possible to produce two copies of |*ψ*_*G*_〉 from a single copy, and hence strictly less can be learned about *G* from a single copy of |*ψ*_*G*_〉 than from a single (coherent) query to the function it encodes.

We conclude our analysis by discussing the security of ${\cal G}_1$ and ${\cal G}_2$ gates. We show that the gate-OTPs we have explored here have strict advantages over any purely classical computational procedure. First, we choose an appropriate figure of merit for which to compare quantum and classical noisy OTPs. An ideal OTP would allow for one, and only one, evaluation of the encoded function, resulting in exactly one input–output pair. We will therefore choose the average probability of evaluating a specific input–output pair correctly, *P*_1_, compared to the average probability of correctness when evaluating all input–output pairs $\tilde P_1$. In the classical case, information can always be copied. Therefore, a classical procedure producing one input–output pair with some fixed probability of success can be repeated arbitrarily many times to produce a noisy version of the encoded gate. The probability of getting a specific input–output pair is equal to the average probability across all input–output pairs, thus $P_1^C = \tilde P_1^C$. However, for ${\cal G}_k$ OTPs this is not the case. If we fix the single line probability of success such that $P_1^C = P_1^Q$, we find that for ${\cal G}_1$ gates $\tilde P_1^Q = 0.75$ while $\tilde P_1^C \approx 0.8536$. Similarly, for ${\cal G}_2$ we find that $\tilde P_1^Q = 0.625$ while $\tilde P_1^C = 0.75$. This shows that our encoding gives an advantage over the best possible classical scheme for an equivalent *P*_1_. Details of these calculations can be found in Supplementary Note 2, where it is also shown that success probability can be boosted via error correction while still maintaining an advantage. Furthermore, in the case of ${\cal G}_1$ gates, we may state that the probability of an adversary finding the parity of two lines, which gives an upper bound on the probability of guessing the complete truth table, is strictly lower than in any possible classical encoding (for details see Supplementary Note 1). This includes noisy implementations of oblivious transfer^25,26^ as our ${\cal G}_1$ gates are equivalent to noisy $$\left( {\begin{array}{*{20}{c}} 1 \\ 2 \end{array}} \right)$$-oblivious transfer. Remarkably, even though oblivious transfer with a vanishing error probability is known to be impossible^26,27^ with our digital signature scheme, we were able to present an implementation whose overall success probability can approach 1.

Aside from the inherent security of an ideal implementation of gate-OTPs, additional measures are necessary in the presence of communication over lossy channels. It is not in general advisable for Alice to simply resend qubits that are not received by Bob, since he can simply claim to have lost a photon to receive a new copy and hence gain additional information about the encoded gate-OTP. This may be prevented via a simple subroutine: for each gate several copies of each state are produced, but each with a randomly chosen additional one-time pad (i.e., a bit flip on the output of all possible inputs). These states are thus in the maximally mixed state as observed from the client and provide no information. Alice will reveal only the one-time pad for the state that Bob confirms to have received and that she wants him to use. Bob will then keep or flip his measurement result, according to Alice’s one-time pad and proceed with the next gate following the same procedure. This subroutine has been used in each of the demonstrated programs. We note that this procedure does necessitate additional interaction between the parties to enable a loss-tolerant implementation of the scheme. Theoretically, the program may be executed at a later point if a non-demolition measurement was utilised to compensate for loss, and quantum memories were employed to maintain the received states. In our implementation, however, it was sufficient to measure the photons at arrival and thus the program was executed directly upon receipt.

## Discussion

Here we have shown the implementation of probabilistic one-time programs in theory and experiment. Our results demonstrate that quantum physics allows for better security trade-offs for certain secure computing tasks than are possible in the classical world, even when perfect security cannot be achieved. This is realised without assumptions on computational hardness, noisy storage or difficulty of entanglement. Using readily available technology we find our results are in good agreement with the theoretical predictions. Future advances in technology that would allow for non-separable measurements on the client’s side could be used to further improve our implementation. We believe the presented work hints at a rich area of quantum protocols to enhance the security of classical computation, even before large-scale quantum computers can be realised.

## Methods

### Experiment

Our single-photon source is based on spontaneous parametric down conversion (SPDC) using a Sagnac loop^28–30^. The pump beam is generated by a 4.5 mW-diode laser at a central wavelength of 394.5 nm, followed by a half- and a quarter-wave plate to adjust the polarisation. It was focused on a 20 mm long, type-II colinear periodically poled Potassium Titanyl Phosphate crystal placed inside the loop, which emitted photon pairs at 789 nm in a separable state |*H*〉 |*V*〉, where *H* and *V* denote horizontal and vertical polarisation, respectively. The down-converted photons were reflected by a dichroic mirror while the pump beam was transmitted. Additionally long-pass and band-pass filters were used to block the pump beam and to select the desired wavelength for the photon pairs. The down-converted photons were then coupled into single-mode fibres and one was directly sent to a detector to herald the second photon. The source was configured in a way that we observed a typical two-photon coincidence rate of 2 kHz with an open switch and the ratio of multi-pair events to single-pair events was <0.07 %. The possibility of multi-pair emissions is a property of every SPDC process which in our case could lead to the transmission of more than one photon at once through the switch and therefore cause unwanted information leaking to the client. Should a future application require even lower (or vanishing) multi-pair emission, this could be implemented using alternative single-photon sources^31–34^.

Furthermore, we implemented an active switch based on a KD*P (potassium dideuterium phosphate) Pockels cell with a half-wave voltage of 6.3 kV and two crossed polarisers. The electronic signal from the avalanche photo diode detector (APD) in the heralding path was sent to a splitterbox which could produce an 'on' and 'off' signal for the driver of the Pockels cell. The pulses were separated by 46 ns which corresponds to the opening time of the switch. During this time voltage is applied to the Pockels cell, causing it to act as a half-wave plate.

These pulses are gated to ensure photons are not transmitted while the LCRs are changing. Once the LCRs are ready to set a state in the program, a gating signal is sent to the splitterbox. Only then will the next heralding signal cause an on/off pulse to be sent to the Pockels cell. All following herald signals will be blocked until the splitterbox receives the next gate signal.

The splitterbox itself causes a delay of the electric signal of 22 ns while the total electronic delay of splitterbox and control electronics is 80 ns. The Pockels cell has a rise-time of 8 ns. To allow for the switch to be opened before the signal photon reaches the Pockels cell in spite of all electronic delays, the signal photon is delayed in a 29 m single-mode fibre. All necessary polarisation states were set using a combination of two LCRs and a QWP at 0°. The maximum time to switch between two states in our scheme was 60 ms. This was therefore the time allowed for every switching process (so as not to leak information about the prepared state because of a shorter switching time). To measure the states in the bases dictated by the inputs to the gates a second set of two LCRs was used followed by a polarising beam splitter and two APDs to measure the photons. Typically 4 % of the times the switch opened a photon was also detected at Bob’s side. This was due to losses in the setup as well as the limited detection efficiency of the APDs. Together with the LCR switching time of 60 ms, this lead to an average gate time of 1.4 s per photon.

### Gates with 1 bit of input

The simplest case of program is one that accepts one bit of input and returns one bit of output. The truth tables for all such ${\cal G}_1$ gates (shown in Fig. 2c) may be easily encoded as:

2 $$\left| {{\mathrm{\Psi }}_0} \right\rangle = \frac{1}{{\sqrt {2 + \sqrt 2 } }}\left( {\left| {0 } \right\rangle+ \left| + \right\rangle } \right),$$

3 $$\left| {{\mathrm{\Psi }}_1} \right\rangle = \frac{1}{{\sqrt {2 + \sqrt 2 } }}\left( {\left| 1 \right\rangle - \left| - \right\rangle } \right),$$

4 $$\left| {{\mathrm{\Psi }}_{{\mathrm{Id}}}} \right\rangle = \frac{1}{{\sqrt {2 + \sqrt 2 } }}\left( {\left| 0 \right\rangle + \left| - \right\rangle } \right),$$

5 $$\left| {{\mathrm{\Psi }}_{{\mathrm{not}}}} \right\rangle = \frac{1}{{\sqrt {2 + \sqrt 2 } }}\left( {\left| 1 \right\rangle + \left| + \right\rangle } \right),$$

$$\left| \pm \right\rangle = \frac{1}{{\sqrt 2 }}\left( {\left| 0 \right\rangle \pm \left| 1 \right\rangle } \right)$$

### Gates with 2 bits of input

${\cal G}_2$ gates can be encoded using either a combination of three states from Eqs. (2)–(5) (which corresponds to the linear scheme) or a combination of two states (elliptical scheme), in which case the above mentioned states need to be combined with additional states from the following list:

6 $$\left| {{\mathrm{\Psi }}_0^e} \right\rangle = \left( { + \frac{1}{2} - \frac{1}{{\sqrt 2 }}i} \right)\left| 0 \right\rangle + \frac{1}{2}\left| 1 \right\rangle ,$$

7 $$\left| {{\mathrm{\Psi }}_1^e} \right\rangle = \left( { - \frac{1}{2} - \frac{1}{{\sqrt 2 }}i} \right)\left| 0 \right\rangle + \frac{1}{2}\left| 1 \right\rangle ,$$

8 $$\left| {{\mathrm{\Psi }}_2^e} \right\rangle = \frac{1}{2}\left| 0 \right\rangle + \left( { + \frac{1}{2} + \frac{1}{{\sqrt 2 }}i} \right)\left| 1 \right\rangle ,$$

9 $$\left| {{\mathrm{\Psi }}_3^e} \right\rangle = \frac{1}{2}\left| 0 \right\rangle + \left( { - \frac{1}{2} + \frac{1}{{\sqrt 2 }}i} \right)\left| 1 \right\rangle ,$$

10 $$\left| {{\mathrm{\Psi }}_4^e} \right\rangle = \left( { + \frac{1}{2} + \frac{1}{{\sqrt 2 }}i} \right)\left| 0 \right\rangle + \frac{1}{2}\left| 1 \right\rangle ,$$

11 $$\left| {{\mathrm{\Psi }}_5^e} \right\rangle = \left( { - \frac{1}{2} + \frac{1}{{\sqrt 2 }}i} \right)\left| 0 \right\rangle + \frac{1}{2}\left| 1 \right\rangle ,$$

12 $$\left| {{\mathrm{\Psi }}_6^e} \right\rangle = \frac{1}{2}\left| 0 \right\rangle + \left( { + \frac{1}{2} - \frac{1}{{\sqrt 2 }}i} \right)\left| 1 \right\rangle ,$$

13 $$\left| {{\mathrm{\Psi }}_7^e} \right\rangle = \frac{1}{2}\left| 0 \right\rangle + \left( { - \frac{1}{2} - \frac{1}{{\sqrt 2 }}i} \right)\left| 1 \right\rangle .$$

The encoding of specific gates is done according to tables shown in the Supplementary Tables 1 and 2. In the linear and elliptical scheme, the gate-encoding state is a tensor product state of three or two photons, respectively. In the linear scheme, each of the three photons are in a state given in Eqs. (2)–(5). As there are 64 combinations and only 16 gates, each gate can be encoded in four different ways (represented by orthogonal state vectors), and a random choice is made each time the gate must be encoded. In the elliptical scheme, the first photon is in a state given in Eqs. (2)–(5), while the second photon is in a state given in Eqs. (6)–(13). As there are 32 combinations and only 16 gates, each gate can be encoded in two different ways, and again a random choice is made each time the gate must be encoded. The random choice between orthogonal state vectors is made by the sender and it is irrelevant from the point of view of the receiver. Thus, the state as seen by the receiver is effectively the mixed state given in Eq. (1).

## Electronic supplementary material

Supplementary Information

## Data Availability

The data that support the findings of this study are available from the corresponding authors upon reasonable request.
