# Supplementary material for: Quantum advantage for probabilistic one-time programs
Source: Nat Commun. 2018 Dec 6;9:5225. doi: 10.1038/s41467-018-07591-2 (PMC6283881; doi:10.1038/s41467-018-07591-2)
Supplement: Supplementary file 1 — Supplementary Information [file 41467_2018_7591_MOESM1_ESM.pdf]

Supplementary Information - Quantum advantage for probabilistic one-time programs

Roehsner and Kettlewell et al.

Gate	Encoding states	Gate	Encoding states
0000	$\begin{cases} \lvert\Psi_0\rangle \otimes \lvert\Psi_0^e\rangle \\ \lvert\Psi_1\rangle \otimes \lvert\Psi_4^e\rangle \end{cases}$	1000	$\begin{cases} \lvert\Psi_{\text{not}}\rangle \otimes \lvert\Psi_0^e\rangle \\ \lvert\Psi_{\text{Id}}\rangle \otimes \lvert\Psi_4^e\rangle \end{cases}$
0001	$\begin{cases} \lvert\Psi_0\rangle \otimes \lvert\Psi_1^e\rangle \\ \lvert\Psi_1\rangle \otimes \lvert\Psi_5^e\rangle \end{cases}$	1001	$\begin{cases} \lvert\Psi_{\text{not}}\rangle \otimes \lvert\Psi_1^e\rangle \\ \lvert\Psi_{\text{Id}}\rangle \otimes \lvert\Psi_5^e\rangle \end{cases}$
0010	$\begin{cases} \lvert\Psi_0\rangle \otimes \lvert\Psi_2^e\rangle \\ \lvert\Psi_1\rangle \otimes \lvert\Psi_6^e\rangle \end{cases}$	1010	$\begin{cases} \lvert\Psi_{\text{not}}\rangle \otimes \lvert\Psi_2^e\rangle \\ \lvert\Psi_{\text{Id}}\rangle \otimes \lvert\Psi_6^e\rangle \end{cases}$
0011	$\begin{cases} \lvert\Psi_0\rangle \otimes \lvert\Psi_3^e\rangle \\ \lvert\Psi_1\rangle \otimes \lvert\Psi_7^e\rangle \end{cases}$	1011	$\begin{cases} \lvert\Psi_{\text{not}}\rangle \otimes \lvert\Psi_3^e\rangle \\ \lvert\Psi_{\text{Id}}\rangle \otimes \lvert\Psi_7^e\rangle \end{cases}$
0100	$\begin{cases} \lvert\Psi_{\text{Id}}\rangle \otimes \lvert\Psi_0^e\rangle \\ \lvert\Psi_{\text{not}}\rangle \otimes \lvert\Psi_4^e\rangle \end{cases}$	1100	$\begin{cases} \lvert\Psi_1\rangle \otimes \lvert\Psi_0^e\rangle \\ \lvert\Psi_0\rangle \otimes \lvert\Psi_4^e\rangle \end{cases}$
0101	$\begin{cases} \lvert\Psi_{\text{Id}}\rangle \otimes \lvert\Psi_1^e\rangle \\ \lvert\Psi_{\text{not}}\rangle \otimes \lvert\Psi_5^e\rangle \end{cases}$	1101	$\begin{cases} \lvert\Psi_1\rangle \otimes \lvert\Psi_1^e\rangle \\ \lvert\Psi_0\rangle \otimes \lvert\Psi_5^e\rangle \end{cases}$
0110	$\begin{cases} \lvert\Psi_{\text{Id}}\rangle \otimes \lvert\Psi_2^e\rangle \\ \lvert\Psi_{\text{not}}\rangle \otimes \lvert\Psi_6^e\rangle \end{cases}$	1110	$\begin{cases} \lvert\Psi_1\rangle \otimes \lvert\Psi_2^e\rangle \\ \lvert\Psi_0\rangle \otimes \lvert\Psi_6^e\rangle \end{cases}$
0111	$\begin{cases} \lvert\Psi_{\text{Id}}\rangle \otimes \lvert\Psi_3^e\rangle \\ \lvert\Psi_{\text{not}}\rangle \otimes \lvert\Psi_7^e\rangle \end{cases}$	1111	$\begin{cases} \lvert\Psi_1\rangle \otimes \lvert\Psi_3^e\rangle \\ \lvert\Psi_0\rangle \otimes \lvert\Psi_7^e\rangle \end{cases}$

Supplementary Table 1: Encoding scheme for $\mathcal{G}_2$ gates, using the two-photon, elliptical scheme. The gates are labelled by the last column of their truth table, e.g. $(00\rightarrow0, 01\rightarrow1, 10\rightarrow0, 11\rightarrow0)$ corresponds to 0100. The quantum states are the qubits referred to in Figure 2f of the main text.

Gate	Encoding states	Gate	Encoding states
0000	$\lvert\Psi_0\rangle \otimes \lvert\Psi_0\rangle \otimes \lvert\Psi_0\rangle$<br>$\lvert\Psi_0\rangle \otimes \lvert\Psi_1\rangle \otimes \lvert\Psi_{\mathrm{Id}}\rangle$<br>$\lvert\Psi_1\rangle \otimes \lvert\Psi_0\rangle \otimes \lvert\Psi_{\mathrm{not}}\rangle$<br>$\lvert\Psi_1\rangle \otimes \lvert\Psi_1\rangle \otimes \lvert\Psi_1\rangle$	1000	$\lvert\Psi_{\mathrm{not}}\rangle \otimes \lvert\Psi_0\rangle \otimes \lvert\Psi_0\rangle$<br>$\lvert\Psi_{\mathrm{not}}\rangle \otimes \lvert\Psi_1\rangle \otimes \lvert\Psi_{\mathrm{Id}}\rangle$<br>$\lvert\Psi_{\mathrm{Id}}\rangle \otimes \lvert\Psi_0\rangle \otimes \lvert\Psi_{\mathrm{not}}\rangle$<br>$\lvert\Psi_{\mathrm{Id}}\rangle \otimes \lvert\Psi_1\rangle \otimes \lvert\Psi_1\rangle$
0001	$\lvert\Psi_0\rangle \otimes \lvert\Psi_{\mathrm{Id}}\rangle \otimes \lvert\Psi_0\rangle$<br>$\lvert\Psi_0\rangle \otimes \lvert\Psi_{\mathrm{not}}\rangle \otimes \lvert\Psi_{\mathrm{Id}}\rangle$<br>$\lvert\Psi_1\rangle \otimes \lvert\Psi_{\mathrm{Id}}\rangle \otimes \lvert\Psi_{\mathrm{not}}\rangle$<br>$\lvert\Psi_1\rangle \otimes \lvert\Psi_{\mathrm{not}}\rangle \otimes \lvert\Psi_1\rangle$	1001	$\lvert\Psi_{\mathrm{not}}\rangle \otimes \lvert\Psi_{\mathrm{Id}}\rangle \otimes \lvert\Psi_0\rangle$<br>$\lvert\Psi_{\mathrm{not}}\rangle \otimes \lvert\Psi_{\mathrm{not}}\rangle \otimes \lvert\Psi_{\mathrm{Id}}\rangle$<br>$\lvert\Psi_{\mathrm{Id}}\rangle \otimes \lvert\Psi_{\mathrm{Id}}\rangle \otimes \lvert\Psi_{\mathrm{not}}\rangle$<br>$\lvert\Psi_{\mathrm{Id}}\rangle \otimes \lvert\Psi_{\mathrm{not}}\rangle \otimes \lvert\Psi_1\rangle$
0010	$\lvert\Psi_0\rangle \otimes \lvert\Psi_{\mathrm{not}}\rangle \otimes \lvert\Psi_0\rangle$<br>$\lvert\Psi_0\rangle \otimes \lvert\Psi_{\mathrm{Id}}\rangle \otimes \lvert\Psi_{\mathrm{Id}}\rangle$<br>$\lvert\Psi_1\rangle \otimes \lvert\Psi_{\mathrm{not}}\rangle \otimes \lvert\Psi_{\mathrm{not}}\rangle$<br>$\lvert\Psi_1\rangle \otimes \lvert\Psi_{\mathrm{Id}}\rangle \otimes \lvert\Psi_1\rangle$	1010	$\lvert\Psi_{\mathrm{not}}\rangle \otimes \lvert\Psi_{\mathrm{not}}\rangle \otimes \lvert\Psi_0\rangle$<br>$\lvert\Psi_{\mathrm{not}}\rangle \otimes \lvert\Psi_{\mathrm{Id}}\rangle \otimes \lvert\Psi_{\mathrm{Id}}\rangle$<br>$\lvert\Psi_{\mathrm{Id}}\rangle \otimes \lvert\Psi_{\mathrm{not}}\rangle \otimes \lvert\Psi_{\mathrm{not}}\rangle$<br>$\lvert\Psi_{\mathrm{Id}}\rangle \otimes \lvert\Psi_{\mathrm{Id}}\rangle \otimes \lvert\Psi_1\rangle$
0011	$\lvert\Psi_0\rangle \otimes \lvert\Psi_1\rangle \otimes \lvert\Psi_0\rangle$<br>$\lvert\Psi_0\rangle \otimes \lvert\Psi_0\rangle \otimes \lvert\Psi_{\mathrm{Id}}\rangle$<br>$\lvert\Psi_1\rangle \otimes \lvert\Psi_1\rangle \otimes \lvert\Psi_{\mathrm{not}}\rangle$<br>$\lvert\Psi_1\rangle \otimes \lvert\Psi_0\rangle \otimes \lvert\Psi_1\rangle$	1011	$\lvert\Psi_{\mathrm{not}}\rangle \otimes \lvert\Psi_1\rangle \otimes \lvert\Psi_0\rangle$<br>$\lvert\Psi_{\mathrm{not}}\rangle \otimes \lvert\Psi_0\rangle \otimes \lvert\Psi_{\mathrm{Id}}\rangle$<br>$\lvert\Psi_{\mathrm{Id}}\rangle \otimes \lvert\Psi_1\rangle \otimes \lvert\Psi_{\mathrm{not}}\rangle$<br>$\lvert\Psi_{\mathrm{Id}}\rangle \otimes \lvert\Psi_0\rangle \otimes \lvert\Psi_1\rangle$
0100	$\lvert\Psi_{\mathrm{Id}}\rangle \otimes \lvert\Psi_0\rangle \otimes \lvert\Psi_0\rangle$<br>$\lvert\Psi_{\mathrm{Id}}\rangle \otimes \lvert\Psi_1\rangle \otimes \lvert\Psi_{\mathrm{Id}}\rangle$<br>$\lvert\Psi_{\mathrm{not}}\rangle \otimes \lvert\Psi_0\rangle \otimes \lvert\Psi_{\mathrm{not}}\rangle$<br>$\lvert\Psi_{\mathrm{not}}\rangle \otimes \lvert\Psi_1\rangle \otimes \lvert\Psi_1\rangle$	1100	$\lvert\Psi_1\rangle \otimes \lvert\Psi_0\rangle \otimes \lvert\Psi_0\rangle$<br>$\lvert\Psi_1\rangle \otimes \lvert\Psi_1\rangle \otimes \lvert\Psi_{\mathrm{Id}}\rangle$<br>$\lvert\Psi_0\rangle \otimes \lvert\Psi_0\rangle \otimes \lvert\Psi_{\mathrm{not}}\rangle$<br>$\lvert\Psi_0\rangle \otimes \lvert\Psi_1\rangle \otimes \lvert\Psi_1\rangle$
0101	$\lvert\Psi_{\mathrm{Id}}\rangle \otimes \lvert\Psi_{\mathrm{Id}}\rangle \otimes \lvert\Psi_0\rangle$<br>$\lvert\Psi_{\mathrm{Id}}\rangle \otimes \lvert\Psi_{\mathrm{not}}\rangle \otimes \lvert\Psi_{\mathrm{Id}}\rangle$<br>$\lvert\Psi_{\mathrm{not}}\rangle \otimes \lvert\Psi_{\mathrm{Id}}\rangle \otimes \lvert\Psi_{\mathrm{not}}\rangle$<br>$\lvert\Psi_{\mathrm{not}}\rangle \otimes \lvert\Psi_{\mathrm{not}}\rangle \otimes \lvert\Psi_1\rangle$	1101	$\lvert\Psi_1\rangle \otimes \lvert\Psi_{\mathrm{Id}}\rangle \otimes \lvert\Psi_0\rangle$<br>$\lvert\Psi_1\rangle \otimes \lvert\Psi_{\mathrm{not}}\rangle \otimes \lvert\Psi_{\mathrm{Id}}\rangle$<br>$\lvert\Psi_0\rangle \otimes \lvert\Psi_{\mathrm{Id}}\rangle \otimes \lvert\Psi_{\mathrm{not}}\rangle$<br>$\lvert\Psi_0\rangle \otimes \lvert\Psi_{\mathrm{not}}\rangle \otimes \lvert\Psi_1\rangle$
0110	$\lvert\Psi_{\mathrm{Id}}\rangle \otimes \lvert\Psi_{\mathrm{not}}\rangle \otimes \lvert\Psi_0\rangle$<br>$\lvert\Psi_{\mathrm{Id}}\rangle \otimes \lvert\Psi_{\mathrm{Id}}\rangle \otimes \lvert\Psi_{\mathrm{Id}}\rangle$<br>$\lvert\Psi_{\mathrm{not}}\rangle \otimes \lvert\Psi_{\mathrm{not}}\rangle \otimes \lvert\Psi_{\mathrm{not}}\rangle$<br>$\lvert\Psi_{\mathrm{not}}\rangle \otimes \lvert\Psi_{\mathrm{Id}}\rangle \otimes \lvert\Psi_1\rangle$	1110	$\lvert\Psi_1\rangle \otimes \lvert\Psi_{\mathrm{not}}\rangle \otimes \lvert\Psi_0\rangle$<br>$\lvert\Psi_1\rangle \otimes \lvert\Psi_{\mathrm{Id}}\rangle \otimes \lvert\Psi_{\mathrm{Id}}\rangle$<br>$\lvert\Psi_0\rangle \otimes \lvert\Psi_{\mathrm{not}}\rangle \otimes \lvert\Psi_{\mathrm{not}}\rangle$<br>$\lvert\Psi_0\rangle \otimes \lvert\Psi_{\mathrm{Id}}\rangle \otimes \lvert\Psi_1\rangle$
0111	$\lvert\Psi_{\mathrm{Id}}\rangle \otimes \lvert\Psi_1\rangle \otimes \lvert\Psi_0\rangle$<br>$\lvert\Psi_{\mathrm{Id}}\rangle \otimes \lvert\Psi_0\rangle \otimes \lvert\Psi_{\mathrm{Id}}\rangle$<br>$\lvert\Psi_{\mathrm{not}}\rangle \otimes \lvert\Psi_1\rangle \otimes \lvert\Psi_{\mathrm{not}}\rangle$<br>$\lvert\Psi_{\mathrm{not}}\rangle \otimes \lvert\Psi_0\rangle \otimes \lvert\Psi_1\rangle$	1111	$\lvert\Psi_1\rangle \otimes \lvert\Psi_1\rangle \otimes \lvert\Psi_0\rangle$<br>$\lvert\Psi_1\rangle \otimes \lvert\Psi_0\rangle \otimes \lvert\Psi_{\mathrm{Id}}\rangle$<br>$\lvert\Psi_0\rangle \otimes \lvert\Psi_1\rangle \otimes \lvert\Psi_{\mathrm{not}}\rangle$<br>$\lvert\Psi_0\rangle \otimes \lvert\Psi_0\rangle \otimes \lvert\Psi_1\rangle$

Supplementary Table 2: Encoding scheme for $\mathcal{G}_2$ gates, using the three-photon, linear scheme. The gates are labelled by the last column of their truth table, e.g. $(00{\to}0, 01{\to}1, 10{\to}0, 11{\to}0)$ corresponds to 0100. The quantum states are the qubits referred to in Figure 2e of the main text.

State	Fidelity
$\vert\Psi_0\rangle$	0.994 $\pm$ 0.006
$\vert\Psi_{\mathrm{Id}}\rangle$	0.995 $\pm$ 0.002
$\vert\Psi_{\mathrm{not}}\rangle$	0.997 $\pm$ 0.005
$\vert\Psi_1\rangle$	0.998 $\pm$ 0.003
$\vert\Psi_0^e\rangle$	0.996 $\pm$ 0.002
$\vert\Psi_1^e\rangle$	0.997 $\pm$ 0.003
$\vert\Psi_2^e\rangle$	0.992 $\pm$ 0.002
$\vert\Psi_3^e\rangle$	0.997 $\pm$ 0.002
$\vert\Psi_4^e\rangle$	0.993 $\pm$ 0.002
$\vert\Psi_5^e\rangle$	0.997 $\pm$ 0.001
$\vert\Psi_6^e\rangle$	0.991 $\pm$ 0.007
$\vert\Psi_7^e\rangle$	0.991 $\pm$ 0.008

Supplementary Table 3: Quantum state fidelity of all used single-qubit states. The error is estimated using a 500-cycle Monte-Carlo simulation with Poissonian noise added to the experimental counts.

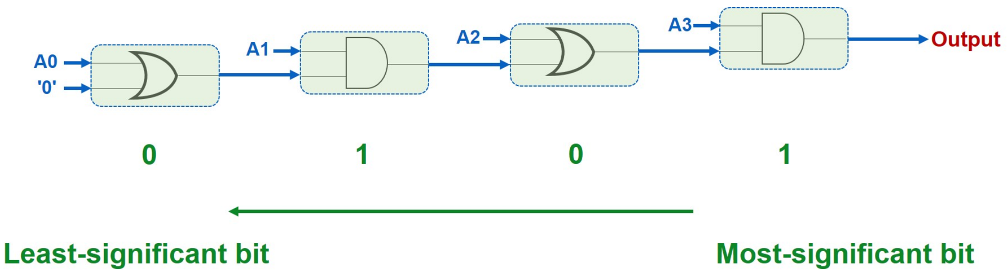

Supplementary Figure 1: Circuit diagram for the implemented solution to the Millionaires Problem. Alice encodes her input in binary representation by sending an OR gate for each bit with the value 0 and an AND gate for each bit with the value 1.

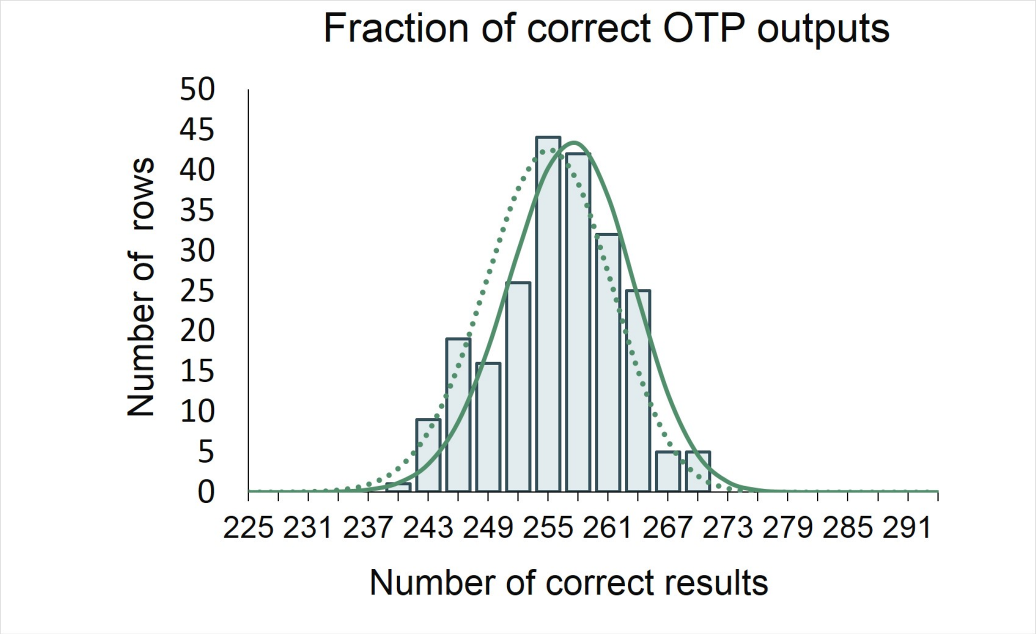

Supplementary Figure 2: The histogram presents the result of the experimental implementation of a delegated digital signature scheme, showing the distribution of number of correct output bits in each row compared to the theoretically expected values when a row length of 300 was used. In the experimental implementation the threshold $\tau = 234$ was chosen as this value maximises the difference between the success probabilities of the honest and dishonest client. It can be seen that this threshold was passed for every row. Considering the expected deviation due to the probabilistic nature of the scheme and experimental imperfections the results are in good agreement with the theoretical predictions. Solid green line: the binomial distribution wherein each $\mathcal{G}_1$ OTP has the theoretically expected success probability for a perfect implementation of probability of correctly giving the correct output. Dotted green line: the binomial distribution based around the average probability of success that could be realised in the experiment (this being slightly reduced compared to the theoretical prediction due to experimental imperfections). Histogram bars are of width 3, with values taken from a single evaluation of a signature.

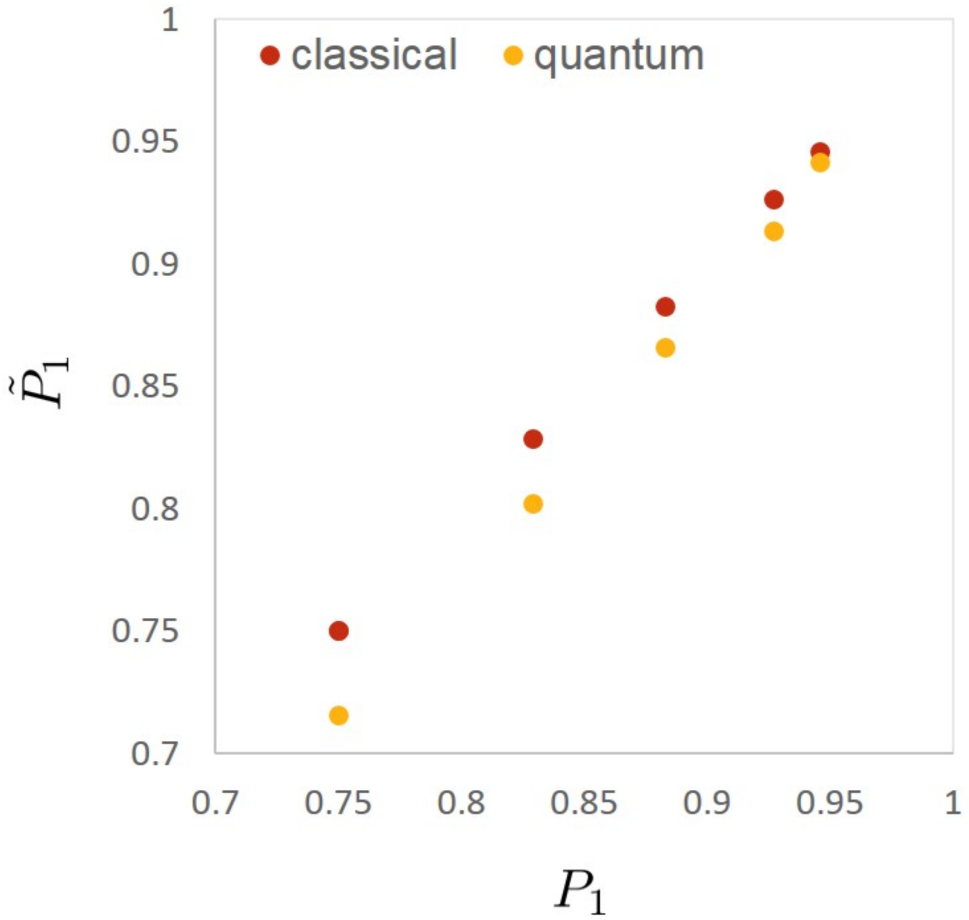

Supplementary Figure 3: The average probability of success when trying to find all lines of a truth table in the quantum case $\tilde{P}_1$ is plotted against the probability of finding exactly one line, P_1, and compared to classical case. The different probabilities of success for a single line could in the quantum case be achieved by using error correction as described in the Supplementary Information. This shows the probability that a single line is correct, for $\mathcal{G}_2$ gates and demonstrates a clear quantum advantage for the noisy one time programs.

Supplementary Note 1 - Advantage over classical encodings for $\mathcal{G}_1$ gates

We will show that the quantum implementation of $\mathcal{G}_1$ gates can hide more information than a classical scheme about the result of multiple lines of a truth table. This will be done by obtaining an inequality which must be satisfied by all classical schemes but is violated by the quantum scheme described in the main text.

We consider a situation where Bob is interested in the parity of some subset of lines of the truth table, which gives us a bound on the probability of identifying these lines in the subset exactly. Using equation 1 of the main text, he considers two states that are formed by summing over all states with equal parity over the subset of lines he is interested in. For subsets consisting of more than one line, these two states are equal and thus impossible to distinguish. In other words, while a single line of the truth table can be found with probability larger than $\frac{1}{2}$, the parity of two or more lines is completely hidden. For comparison, a classical scheme that encodes $\mathcal{G}_1$ gates with single-line error probability higher than $3/4$ must give correct results about the parity of two lines with probability higher than $1/2$. In particular, if the single-line error probability is $\frac{1}{2}+\frac{1}{2\sqrt{2}}$ (the same as achieved with the quantum states in equation 1 of the main text), the classical scheme must allow the parity of two lines to be correctly identified with probability at least $1/\sqrt{2}$, which is greater than $1/2$.

In order to improve the probability of getting the correct output from a particular gate, the programmer may send multiple copies, c, of the state corresponding to this gate. The client is expected to make some (possibly non-local) multi-qubit measurement to evaluate the line of the truth table corresponding to their input. In this case the statement that the parity of multiple lines of the truth table of the encoded gates are perfectly hidden is no longer valid. Sending multiple copies of the state in equation 1 of the main text creates a trade-off situation between precision and security, where precision is quantified by the success probability that an honest client can achieve when evaluating a single line, and security is quantified by the amount of information that can be found about multiple lines of the truth table simultaneously. A complete lack of security occurs when the client can perfectly identify which one of the gates is represented by the quantum state he possesses.

We will now make a comparison between a quantum scheme and what could be achieved by a classical scheme. Any classical scheme encoding a gate can be repeatedly rerun to generate a noisy truth table for the

encoded gate. We will see that these noisy truth tables must satisfy an inequality that bounds the maximum level of security that can be achieved for a given level of precision. This inequality is violated in the quantum case, allowing us to achieve more security for the same level of precision than any classical scheme.

Analytical results - Classical $\mathcal{G}_1$

Without loss of generality, we consider a classical model in which a programmer introduces some errors in the gate truth table. These are errors purposefully introduced at compile time. There is no point in introducing random errors at run time, since the client can evaluate the truth table multiple times and find the most common value with high probability. If there is some anti-correlation in the presence of errors in different lines, then the probability of getting a second line correct is decreased when conditioned on getting the first line correct.
To obtain this anti-correlation we consider that the programmer introduces h errors in the truth table (with $0 \leq h \leq 2$) with probability E_h (so $E_0 + E_1 + E_2 = 1$). If one error is introduced it can affect either line with equal probability. Thus for an honest client interested in a single line of the truth table, the average probability that the obtained result is correct is

$$F_1^{\mathsf{C}} = E_0 + \frac{1}{2}E_1 \tag{1}$$

Meanwhile, for a dishonest client interested in the parity of both lines, the probability that the obtained result is correct is

$$F_2^{\mathsf{C}} = E_0 + E_2 \tag{2}$$

We can invert these equations to find E_h in terms of F_1^{C} and F_2^{C}. This tells the programmer what is the probability distribution in the number of errors that need to be introduced in order to produce an encoding that is characterized by given values of the probability of decoding a single line and of decoding the parity of both lines. This leads to the result

$$E_0 = F_1^{\mathsf{C}} + \frac{1}{2}F_2^{\mathsf{C}} - \frac{1}{2} \tag{3}$$

$$E_1 = 1 - F_2^{\mathsf{C}} \tag{4}$$

$$E_2 = \frac{1}{2} - F_1^{\mathsf{C}} + \frac{1}{2}F_2^{\mathsf{C}} \tag{5}$$

Each of these terms must be non-negative, which is only possible if

$$F_2^{\mathsf{C}} \geq \left|2F_1^{\mathsf{C}} - 1\right| \tag{6}$$

This means that an attempt at a classical noisy gate which outputs correct results with probability F_1^{C} also allows one to probe the parity of both lines of its truth table with probability greater than $\left|2F_1^{\mathsf{C}} - 1\right|$. However, we will see that a quantum implementation of the noisy OTPs violate this inequality, showing that it hides more information about other lines of the truth table than is possible classically.

Analytical results - Quantum $\mathcal{G}_1$

The figure of merit that we consider for security in this section is F_h, the probability of success in calculating the parity of a subset of the lines of the truth table, as a function of the size h of this subset (with $1 \leq h \leq 2$ in the case of $\mathcal{G}_1$ gates, whose truth table has only two lines). The outcome of the parity determination is binary, so we can use known results on quantum state discrimination of two quantum states. Specifically, the optimal probability of distinguishing them is uniquely determined by the 1-norm of half of their difference. For an honest client who is interested in only the first line of the truth table, the probability of success is related to the 1-norm of the operator

$$\hat{A}_1 = \frac{1}{4}\rho_{00}^{\otimes c} + \frac{1}{4}\rho_{01}^{\otimes c} - \frac{1}{4}\rho_{10}^{\otimes c} - \frac{1}{4}\rho_{11}^{\otimes c} \tag{7}$$

while for a dishonest client who is interested in obtaining the parity of both lines, the probability of success is related to the 1-norm of the operator

$$\hat{A}_2 = \frac{1}{4}\rho_{00}^{\otimes c} - \frac{1}{4}\rho_{01}^{\otimes c} - \frac{1}{4}\rho_{10}^{\otimes c} + \frac{1}{4}\rho_{11}^{\otimes c} \tag{8}$$

Gates with one bit of input may be encoded as pure states, so providing multiple copies of them does not increase the dimensionality of the effective Hilbert space, which is spanned by at most four linearly independent vectors. For simplicity, we consider the case where the number of copies is odd and obtain the following results

$$F_1^{\mathrm{Q}} \equiv \frac{1}{2} + \frac{1}{2}\left\|\hat{A}_1\right\|_1 = \frac{1}{2} + \frac{1}{2}\sqrt{1 - \frac{1}{2^c}} \tag{9}$$

$$F_2^{\mathrm{Q}} \equiv \frac{1}{2} + \frac{1}{2}\left\|\hat{A}_2\right\|_1 = \frac{1}{2} + \frac{1}{2}\sqrt{1 - \frac{2}{2^c}} \tag{10}$$

where the superscript Q refer to a quantum implementation. These values do not satisfy the inequality in Supplementary Equation 6. This means that, if we compare a classical scheme which offers the same level of precision for an honest client (i.e., $F_1^{\mathrm{C}} = F_1^{\mathrm{Q}}$), the probability of success for a dishonest client is higher in the classical case. Similarly, if we restrict the two protocols to the same level of security as quantified by the probability of finding the parity of both lines, then the quantum OTP can offer better performance for honest clients than any classical scheme.

Supplementary Note 2 - Advantage over classical encodings for other gates

We demonstrate the advantage of the quantum one-time programs with multiple bits of input over possible classical schemes. We assume that the gate-OTP is *a priori* equally likely to encode any of the possible gates in $\mathcal{G}_k$. Although we will focus on $\mathcal{G}_2$ gates, parts of this discussion can be generalized to $\mathcal{G}_k$ gates with $k > 1$.
We consider the probability distribution for (potentially correlated) Bernoulli random variables X_i, which are equal to 1 if and only if a query to the a noisy classical truth table encoding gate G for input i returns $G(i)$. All probability distributions over truth tables can be described in this way, and so it can be used to obtain a bound on the trade-offs inherent in any classical scheme. The sender does not know in advance which lines of the truth table the client might be interested, thus his/her interest is in minimizing the worst-case probability of correctly obtaining the output for multiple lines across all sets of lines. To do that, every line is treated in an equivalent way, and so all the elements on the diagonal of the covariance matrix of the Bernoulli variables X_i will be equal, as will be all off-diagonal elements. The covariance matrix thus has the form $u\mathbb{I} - v\mathbb{M}$, where $\mathbb{I}$ is the identity matrix and $\mathbb{M}$ is the matrix with all entries equal to one. In order to obtain a fixed probability P_1 of correctness for a single query to a line of the truth table, it must be the case that $u - v = P_1 - P_1^2$. Furthermore, since the minimum eigenvalue of such a matrix is $u - 2^k v$ and covariance matrices are positive semi-definite, it must be the case that $u - v \geq (2^k - 1)v$.

With these arguments, it's possible to bound the probability of obtaining the correct values for two lines (indexed by x and y, with $x \neq y$) of such a truth table,

$$\begin{aligned}\tilde{P}_2 &= E(X_x X_y)\\ &= E((X_x - P_1)(X_y - P_1)) + P_1^2\\ &= -v + p^2\\ &\geq -\frac{(u-v)}{2^k - 1} + P_1^2\\ &= \frac{P_1^2 - P_1}{2^k - 1} + P_1^2\\ &= \frac{2^k P_1^2 - P_1}{2^k - 1}\end{aligned} \tag{11}$$

As the probability of evaluating a single line of a $\mathcal{G}_2$ OTP is $P_1 = 0.75$, a noisy classical truth table with the same success probability gives $\tilde{P}_1 = 0.75$ across all lines. The probability of correctly decoding a pair of lines is at least $\tilde{P}_2 = 0.5$, independently of the chosen pair of lines.

We may now compare this to the average probability of finding the output values of encoded gates for pairs of inputs. In the quantum case, if the client is interested in a particular line of the truth table, it's possible to implement a quantum measurement strategy that is specifically tuned to increase the probability of getting this value correctly. However, this degrades the available information about the other lines. Thus, in a marked difference to the classical scheme, the probability P_1 of finding the correct value of a particular line is different (and higher) than the average probability $\tilde{P}_1$ of getting a line correctly when the client is trying to identify the whole truth table. The same argument is valid when the client is interested in a given pair of lines, as compared to the average probability of correctly identifying pairs of lines when trying to identify the whole truth table.

Using the quantum encoding without error correction, a client interested in a given line of the truth table of a $\mathcal{G}_2$ gate can correctly identify it with probability equal to 0.75. On the other hand, when the client tries to identify the whole truth table, the average success probability is only 0.625. Looking at pairs of lines, making a specific measurement can allow the client to obtain a probability of success equal to 0.5, but the average over all lines in a measurement of all lines is only 0.375.

Supplementary Note 3 - Optimal measurements

We turn our attention to the measurement strategy that a dishonest client could follow if he is interested in identifying all lines of the truth table of an encoded gate G. This problem is cast as a quantum state discrimination of one state among 2^k alternatives, and the figure of merit is the probability of making a correct guess about the entirety of the truth table. We will consider the “pretty good measurement” strategy introduced by Hausladen and Wootters [1] and another strategy introduced by Ježek, Reháček and Fiurášek [2]. Because of some properties of the way that the gates are encoded in quantum states, these two strategies are equal and also optimal, because they obey established criterias [3].
Considering an ensemble of states $\{\rho_i\}$ with a priori distributions $\{q_i\}$, there are several good measurement strategies for distinguishing them. One candidate strategy is the pretty good measurement, or $\mathcal{PGM}$ [1], for which the POVM corresponding to an output x (where x is a 2^k-bit string representing the truth table) is

$$M_x^{\mathcal{PGM}} = \left(\sum_{s=1} q_s \rho_s\right)^{-1/2^+} q_x \rho_x \left(\sum_{l=s} q_s \rho_s\right)^{-1/2^+} \tag{12}$$

where the operation $A^{-1/2^+}$ is defined as

$$A^{-1/2^+} = \sum_{j:a_j>0} a_j^{-1/2} \left|a_j\right\rangle \left\langle a_j\right| \tag{13}$$

with a_j and $|a_j\rangle$ being the eigenvalues and eigenvectors of A. In a similar manner, slightly more complex sets of measurement operators may be formed that are called the Ježek-Reháček-Fiurášek iterative measurement operators [2]. These are defined recursively, where each iteration is indexed by w.

$$M_x^{\mathcal{JRF},w} = \left(\sum_{s=1} q_s^2 \rho_s M_s^{(w-1)} \rho_s\right)^{-1/2^+} q_x^2 \rho_x M_x^{(w-1)} \rho_x \left(\sum_{s=1} q_s^2 \rho_s M_s^{(w-1)} \rho_s\right)^{-1/2^+} \tag{14}$$

where in the first iteration $M_x^{\mathcal{JRF},0} = \mathbb{I}/2^k$.
Due to the form of our states, each satisfy $\rho_x^2 = \xi \rho_x,\ \forall x$, where ξ is a proportionality constant independent of x. Coupled with an assumption

that the states are *a priori* equiprobable (so that $q_x = \zeta,\ \forall x$, where ζ is a constant independent of x), the $\mathcal{PGM}$ operators are equal to every iteration of the $\mathcal{JRF}$ operators.
It is known that a POVM strategy is optimal if it satisfies the following two conditions [3],

$$M_x\left(q_x\rho_x - q_y\rho_y\right)M_y = 0\ ,\forall x, y \tag{15}$$

$$\sum_{x=0}^{2^k-1} q_x\rho_x M_x - q_y\rho_y \succeq 0\ ,\forall y \tag{16}$$

In a numerical study, we have verified that the $\mathcal{PGM}$ strategy is optimal when ρ_x represents three or less copies of the gate-encoding states.

Optimality of the measurement for single copies

We now give an analytical proof that the $\mathcal{PGM}$ strategy is optimal when a single copy of the quantum states is sent. First we note that, in the case of a single copy, $\sum_s q_s\rho_s = \mathbb{I}$, assuming as before that the states are *a priori* equiprobable (when the number of copies is larger than one, then $\sum_s p_s\rho_s \neq \mathbb{I}$ even in the equiprobable case). Thus, the measurement operators M_x are proportional to the density matrices ρ_x.
We will now obtain a bound on the value of tr$\left(\rho_x Mxi\right)$ using Hö lders inequality,

$$\|fg\|_1 \leq \|f\|_p\|g\|_q \tag{17}$$

which is valid when $\frac{1}{p}+\frac{1}{q} = 1$. Assuming that each state is equally probable and the normalization condition $\sum_x M_x = \mathbb{I}$, this implies that tr$\left(M_x\right) = D/2^k\ ,\forall x$. We also note that $M_x \succeq 0$ as required for POVMs. Using the values $p = 1$, $q = \infty$, $f = M_x$ and $g = \rho_x$ in Hölders inequality, we find that

$$\begin{aligned}|\text{tr}(M_x\rho_x)| &= \|M_i x\rho_x\|_1 \\ &\leq \|M_x\|_1\,\|\rho_x\|_\infty \\ &= \frac{D}{2^k}\frac{2}{D} \\ &= 2^{1-k}\end{aligned} \tag{18}$$

We now use the POVM given by the $\mathcal{PGM}$ (or $\mathcal{JRF}$) operators and show that this saturates the above inequality.

$$\begin{aligned}\text{tr}(M_x\rho_x) &= \sum_j u_j r_j \\ &= \sum_{j:u_j,r_j\neq 0} u_j \frac{2}{D} \\ &= \text{tr}(M_x)\frac{2}{D} \\ &= \frac{D}{2^k}\frac{2}{D} \\ &= 2^{1-k}\end{aligned} \tag{19}$$

where u_j and r_j are the eigenvalues of M_x and ρ_x respectively and we have used the fact that, because $M_x \propto \rho_x$, they both have degenerate eigenvalues in the same eigenbasis. As the $\mathcal{PGM}$/$\mathcal{JRF}$ measurement achieves the exact upper bound on $|\text{tr}(\rho_x M_x)|$, they must be optimal.

Applying the optimal measurements

We now look at situation where the client uses these operators to try to learn what state was sent. We may quantify the number of lines of the truth table the client can on average obtain correctly. We define E_h as the probability that *exactly* h lines are incorrect; in other words, h is the Hamming distance between the encoded gate and the result of a measurement that tries to identify the gate. We consider the average taken over all gates in $\mathcal{G}_k$, and hence over all ρ_x,

$$E_h = \sum_x q_x \sum_{s:\mathcal{H}(s,x)=h} \text{tr}\left(\rho_x M_s\right) \tag{20}$$

where $\mathcal{H}\left(s,x\right)$ is the Hamming distance between the truth tables represented by s and x. From this average number of errors, we can then consider the average probability of correctly identifying a subset of L lines, $\tilde{P}_L$, which is given by

$$\tilde{P}_1 = E_0 + \frac{\binom{3}{1}}{\binom{4}{1}} E_1 + \frac{\binom{2}{1}}{\binom{4}{1}} E_2 + \frac{\binom{1}{1}}{\binom{4}{1}} E_3 = E_0 + \frac{3}{4} E_1 + \frac{1}{2} E_2 + \frac{1}{4} E_3 \tag{21}$$

$$\tilde{P}_2 = E_0 + \frac{\binom{3}{2}}{\binom{4}{2}} E_1 + \frac{\binom{2}{2}}{\binom{4}{2}} E_2 = E_0 + \frac{1}{2} E_1 + \frac{1}{6} E_2 \tag{22}$$

$$\tilde{P}_3 = E_0 + \frac{\binom{3}{3}}{\binom{4}{3}} E_1 = E_0 + \frac{1}{4} E_1 \tag{23}$$

$$\tilde{P}_4 = E_0 \tag{24}$$

In Supplementary Figure 3, $\tilde{P}_1$ in the quantum case is plotted against $\tilde{P}_1$ in the classical case, which is simply the probability that a single line is correct, for $\mathcal{G}_2$ gates. This shows a clear quantum advantage for noisy one time programs.

Supplementary Note 4 - Description of the Private Key Signature scheme

This scheme allows Alice to delegate to Bob the power of digitally signing a message of his choice once and only once. To realize this, Alice's digital signature will be formed by the output of one-time programs. These OTPs take Bob's message as an input and output a valid signature. To allow the signing algorithm to work on a fixed-size input Bob creates a hash of his message using SHA3-224 protocol (there is no particular theoretical reliance on this or any particular hash, but we chose to use SHA3-224 in our demonstration). The signature is verified by Alice, the programmer, by comparing the generated signature against the ideal one that would be produced in the case of perfect OTPs. For each bit of the hash the client is provided with T OTPs, each of which is chosen uniformly at random from the set of $\mathcal{G}_1$ OTPs (in principle we could use $\mathcal{G}_k$ gates, but we chose to use $k = 1$ in our demonstration). The client makes measurements on these states according to the corresponding bit of his hash, producing an array where each row corresponds to the output bits for a single hash bit. The signature is deemed to pass if each row is correct in at least τ places, wherein the threshold τ is a integer predetermined by the programmer. We will show now how the scheme displays a clear example of a situation where even probabilistic OTPs may be used to implement a program which works with a high probability of success.

We compare the probability of success of passing the verification step for

an honest client signing one message to the probability of passing the verification step twice for a dishonest client signing two messages which hash to different values. We will consider the cases where the hashes differ by only one bit. This is a worst case scenario in which an adversary has the maximum probability of cheating successfully. The threshold value τ is chosen to maximise the difference between the success probabilities for an honest and a dishonest client in such a case.

Probability that a dishonest client can pass the verification step for a single bit of the hash :The two signatures taken together constitute a string of lenght $2T$. Each signature needs to be correct in at least τ places to pass the verification stage and thus a necessary (but not sufficient) condition for the combined string to pass is that it matches the concatenation of the two ideal signatures in 2τ places. We place an upper bound on the probability of this happening by using a similar method to that used by Vazirani [4]. The two ideal signatures are encoded in T qubits as is the case when we are sending T $\mathcal{G}_1$ OTPs. It's considered that each of the $2T$-bit strings corresponding to possible signatures is mapped to a pure state $|\phi_x\rangle$, while a measurement that would output a $2T$-bit string y is associated with a projector P_y. This can be done without loss of generality since the measurement projectors can be defined in a larger Hilbert space than the received OTP state, since $|\phi_x\rangle$ may contain an arbitrary number of additional ancilla qubits. The probability that at most h mistakes are made in such a decoding protocol is given by

$$\begin{aligned}\mathcal{P} &\equiv \text{Prob}\left(H(x,y) \leq h\right) \\ &= \frac{1}{2^{2T}} \sum_{x,y:H(x,y)\leq h} \text{tr}\left(P_y\,|\phi_x\rangle\langle\phi_x|\right)\end{aligned} \tag{25}$$

where $H(x,y)$ is the Hamming distance between the strings x and y.
At this moment it is helpful to analyse some properties of the specific ways in which the $|\phi_x\rangle$ states are defined. The $2T$ bits of x are split in pairs corresponding to the i-th bit of each signature, and each pair is encoded in a qubit using the model for $\mathcal{G}_1$ gate-OTPs. Thus, all $|\phi_x\rangle$ states can be written as

$$|\phi_y\rangle = |\phi_{y_1}\rangle \otimes |\phi_{y_2}\rangle \otimes \cdots \otimes |\phi_{y_T}\rangle \otimes |\mathcal{A}\rangle \tag{26}$$

where $y_k \in \{00, 01, 10, 11\}$ and $|\mathcal{A}\rangle$ represents the state of an arbitrary-dimensional ancilla, which does not depend on y. Two states $|\phi_x\rangle$ and $|\phi_y\rangle$

are orthogonal if there is at least one pair of bits (which are encoded in the same qubit) which differ between x and y in both bits. This suggests a way to find an orthonormal basis for this space, by starting with any $|\phi_y\rangle$ and obtain other states by negating pairs of bits from y. Since there are T pairs to negate and all states obtained this way are orthogonal to each other, they form a orthonormal basis with 2^T elements. Given that the space spanned by possible OTP states is of dimension 2^T, and that every state can be written as a linear combination of some others, this basis must span the space generated by all $|\phi_y\rangle$ states. We call this the y-basis. Using these properties, we argue that that the operator $\sum_{x:H(x,y)\leq h} |\phi_x\rangle\langle\phi_x|$ is diagonal in the y-basis just defined, and that $|\phi_y\rangle$ is the eigenvector corresponding to its largest eigenvalue. To see this, we need to consider what the strings x appear in the sum. Specifically, for each string x where the first bit of a given pair does not match the corresponding bit in y (but the second bit of that pair does match), there is also another string where the first bit matches but the second bit does not match. These strings are always both included or both excluded, because the Hamming distance between each of them and y is the same. The mixture associated with these two states is diagonal in the y basis, even though none of them are individually.

With the eigenvectors already found, the task is to find eigenvalues. For an eigenvector $|\phi_z\rangle$, the eigenvalue depends on how many strings x that are not orthogonal to z are included in the summation. Because the summation over x is centered around y (in the sense of the Hamming distance), the eigenvector $|\phi_y\rangle$ has the highest number of strings x appearing in the sum. This leads to this eigenvalue being the highest one. By a counting argument, it's possible to arrive at its specific value. Strings x that appear in the sum are at Hamming distance at most h from y, but if both bits of a given pair are different in x and y then the state corresponding to this string does not contribute. If a pair is equal in x and y, then the contribution to $\langle\phi_y \mid \phi_x\rangle \langle\phi_x \mid \phi_y\rangle$ corresponding to that qubit is 1. If a pair has x and y differing in one bit, the contribution to $\langle\phi_y \mid \phi_x\rangle \langle\phi_x \mid \phi_y\rangle$ is $1/2$, but because there are two of those states, their sum also constributes 1. Thus, when a Hamming distance of w between x and y is considered, we must consider only terms where there is either zero or one differences per pair, with each configuration contributing 1. The eigenvalue corresponding to $|\phi_y\rangle$ is then

$$\lambda = \sum_{w=0}^{h} \begin{pmatrix} T \\ w \end{pmatrix} \tag{27}$$

This was explicitly checked for small values of T by numerical diagonalization.
We can now find an upper bound to the probability Prob $(H(x,y) \leq h)$ that a dishonest client can make at most h mistakes in the determination of the $2T$-bit string corresponding to the ideal signatures for two distinct messages. Continuing from Supplementary Equation 25, we have that

$$\mathcal{P} = \frac{1}{2^{2T}} \sum_y \text{tr}\left(P_y \sum_{x:H(x,y)<=h} |\phi_x\rangle\langle\phi_x| \right)$$
$$\leq \frac{1}{2^{2T}} \sum_y \text{tr}\left(P_y Q\right) \sum_{w=0}^{h} \binom{T}{w} \tag{28}$$

where Q is a projector to the codespace spanned by the codewords $|\phi_x\rangle$, which has dimension 2^T. Then,

$$\mathcal{P} \leq \frac{1}{2^{2T}} \text{tr}\left(\left(\sum_y P_y\right) Q\right) \sum_{w=0}^{h} \binom{T}{w}$$
$$= \frac{1}{2^{2T}} \text{tr}\left(Q\right) \sum_{w=0}^{h} \binom{T}{w}$$
$$= \frac{1}{2^{T}} \sum_{w=0}^{h} \binom{T}{w} \tag{29}$$

If $h/T < (1/2) - \epsilon$, for any positive constant ϵ, the probability of obtaining an output string within Hamming distance h of the ideal signature string is exponentially small in T. Returning to the definition of h as $2T - 2\tau$, we see that the exponential suppression happens when the ratio τ/T is fixed as any constant greater than $3/4$.
We now have an upper bound for the probability of success of a dishonest client passing the verification step for a single bit of the hash for two different inputs. As we assume a worst case scenario, where the hashes differ in only a single bit the client can follow the honest scenario for all other bits of his hash. Therefore, the overall probability of a dishonest client to sign two such messages is simply given by the product of the individual success probabilities per bit.
It becomes increasingly unlikely that the client is able to sign two messages if the required threshold for signing one message is set as a constant fraction $\alpha > 3/4$ of T. If the threshold τ is set at lower than $\left(\frac{1}{2} + \frac{1}{2\sqrt{2}}\right) T \approx$

$(0.85) \cdot T$, the honest client is able to sign a single message with probability that approaches 1 as T is increased.
In conclusion, when the threshold τ is chosen to lie between $(0.75) \cdot T$ and $(0.85) \cdot T$, a client can sign one message with high probability but can sign two messages with low probability. In the limit of high T, these probabilities tend to 1 and 0, respectively. For practical reasons, as a trade-off between security and speed, we chose the values $T = 300$ and $\tau = 234$, which results in a client being able to sign one message with probability 97%, but with a smaller than 4% probability of signing two messages which hashes to strings differing in only one bit. This is an upper bound to the cases where the hashes are different in more than one bit. Another interesting feature of the protocol is that it does not require a perfect implementation of the quantum states. Noise can be tolerated as long as the probability of obtaining a correct outcome for a single line of the $\mathcal{G}_1$ OTP is higher than 75%, provided that τ is chosen accordingly and T is high enough such that the client can sign one message with reasonably high probability.

Supplementary References

[1] Hausladen, P. & Wootters, W. K. A pretty good measurement for distinguishing quantum states. *Journal of Modern Optics* **41**, 2385–2390 (1994).

[2] Ježek, M., Řeháček, J. & Fiurášek, J. Finding optimal strategies for minimum-error quantum-state discrimination. *Physical Review A* **65**, 060301 (2002).

[3] Bae, J. & Kwek, L.-C. Quantum state discrimination and its applications. *Journal of Physics A: Mathematical and Theoretical* **48**, 083001 (2015).

[4] Vazirani, U. Quantum random access codes and applications. `https://people.eecs.berkeley.edu/~vazirani/s09quantum/notes/lecture10.pdf` (2009).
